# First-episode psychosis and substance use in an acute mental health unit

**DOI:** 10.4102/sajpsychiatry.v24i0.1276

**Published:** 2018-09-20

**Authors:** Yanga Thungana, Zukiswa Zingela, Stefan van Wyk

**Affiliations:** 1 Department of Psychiatry, Walter Sisulu University, South Africa

## Abstract

**Background:**

Substance use is frequent among first-episode psychosis (FEP) patients, and it has prognostic implications. There is a dearth of locally published literature in the Eastern Cape, South Africa, on the prevalence of substance use amongst FEP patients, especially among inpatient populations. This study has been undertaken to fill this gap with the aim being to yield further knowledge which could be applicable to the Eastern Cape psychiatric inpatient population. The research site is a 35-bed designated mental health unit (MHU) in a regional hospital in Port Elizabeth, South Africa.

**Method:**

This was a cross-sectional, retrospective and descriptive study. Case notes of patients admitted to the MHU with first-episode, non-affective psychosis over a 12-month period – from November 2016 to October 2017 – were analysed using a pre-designed data capturing sheet to gather demographic, clinical and other relevant information. A total of 331 patients were identified to have presented with first time psychosis and 206 of them were excluded based on the inclusion and exclusion criteria.

**Results:**

Of the 331 cases, 125 met the inclusion criteria. The majority of those presenting with FEP were predominantly males (92 of 125, 73.6%). The median age of the 125 participants was 25 years with a mean age of 28 years (SD 11), and 74% aged between 18 and 35 years. The majority of the 125 subjects were unemployed (62%) and single (84%), with only high school education or less (81%), and were brought to hospital by family members (57%). Of the 125 participants presenting with FEP, 83% had a current or lifetime history of substance use. Cannabis was the most commonly used substance (64%), followed by alcohol (60%), stimulants (42%), mandrax (12.5%) and opioids (7.5%). Cannabis use was associated with a younger age (mean 25 years, SD 7) compared to alcohol (mean 29 years, SD 11) use. The cannabis and stimulant use were disproportionally higher in males with FEP (76% and 47%, respectively vs. 26% and 23% in females) compared to alcohol use (62% in males vs. 55% in females).

**Conclusion:**

In keeping with national and international literature, the study results showed a strong association between FEP and a history of current or lifetime substance use. The strongest association is with the use of cannabis and alcohol followed by stimulants. The study findings highlight the need for mental health services in the Eastern Cape to focus on dual diagnosis in order to address the challenge of substance abuse and its association with FEP. Preventative strategies focusing on substance use disorder could also assist in addressing the growing burden of mental disorders.

